# The influence of monoacylglycerol lipase inhibition upon the expression of epidermal growth factor receptor in human PC-3 prostate cancer cells

**DOI:** 10.1186/1756-0500-7-441

**Published:** 2014-07-10

**Authors:** Mariateresa Cipriano, Sandra Gouveia-Figueira, Emma Persson, Malin Nording, Christopher J Fowler

**Affiliations:** 1 Department of Pharmacology and Clinical Neuroscience, Umeå University, Umeå, Sweden; 2 Department of Chemistry, Umeå University, Umeå, Sweden; 3 Department of Radiation Sciences, Oncology, Umeå University, Umeå, Sweden

**Keywords:** Prostate cancer, Epidermal growth factor, Cannabinoid receptor, Monoacylglycerol lipase

## Abstract

**Background:**

It has been reported that direct activation of the cannabinoid CB_1_ receptor in epidermal growth factor (EGR)-stimulated PC-3 prostate cancer cells results in an anti-proliferative effect accompanied by a down-regulation of EGF receptors (EGFR). In the present study, we investigated whether similar effects are seen following inhibition of the endocannabinoid hydrolytic enzyme monoacylglycerol lipase (MGL).

**Results:**

CB_1_ receptor expression levels were found to differ greatly between two experimental series conducted using PC-3 cells. The monoacylglycerol lipase inhibitor JZL184 increased levels of 2-arachidonoylglycerol in the PC-3 cells without producing changes in the levels of anandamide and related *N*-acylethanolamines. In the first series of experiments, JZL184 produced a small mitogenic effect for cells that had not been treated with EGF, whereas an anti-proliferative effect was seen for EGF-treated cells. An anti-proliferative effect for the EGF-treated cells was also seen with the CB receptor agonist CP55,940. In the second batch of cells, there was an interaction between JZL184 and CB_1_ receptor expression densities in linear regression analyses with EGFR expression as the dependent variable.

**Conclusions:**

Inhibition of MGL by JZL184 can affect EGFR expression. However, the use in our hands of PC-3 cells as a model to investigate the therapeutic potential of MGL inhibitors and related compounds is compromised by their variability of CB_1_ receptor expression.

## Background

It is now well established that ligands activating cannabinoid (CB) receptors, can affect the viability of a variety of different cancer cells lines [1]. With respect to prostate cancer (Pca) cells, Nithipatikom *et al.*[2] reported that compounds reducing the synthesis of the endocannabinoid ligand 2-arachidonoylglycerol (2-AG) increased the invasivity of the cells *in vitro*, whereas the reverse was seen when the metabolism of this endocannabinoid was blocked. A subsequent study reported that a selective blockade of the primary 2-AG metabolising enzyme monoacylglycerol lipase (MGL), either by use of the selective MGL inhibitor JZL184 or by shRNA knockdown of the enzyme, affected cell survival and invasion *in vitro* and reduced tumour growth in a xenograft model [3]. This effect was due in part to activation of CB_1_ receptors by the increased 2-AG concentration, and in part to blockade of the production of long chain fatty acids by the MGL-catalysed hydrolysis of the corresponding monoacylglycerols [3]. Fatty acid amide hydrolase (FAAH), the enzyme responsible for hydrolysis of the other main endocannabinoid anandamide (AEA), is also overexpressed in Pca [4,5] and transfection of cultured Pca cells with this enzyme increases their invasivity [4].

Taken together, the studies described above suggest that the endocannabinoid system can act as a local regulatory mechanism to keep Pca cells in check and that MGL is a potential therapeutic target. The mechanism(s) by which activation of CB receptors affect Pca cell survival are a matter of current research [1], but one important route is via a down-regulation of the receptors for epidermal growth factor (EGF) [6]. These receptors (EGFR) are involved in the regulation of cell growth and survival, and overexpression of their phosphorylated (active) form is associated with a poor disease-specific survival [7]. Mimeault and colleagues [6] showed that treatment of Pca cells, including PC-3 androgen-independent Pca cells with AEA reduced the expression of EGFR in a manner blocked by the CB_1_ receptor antagonist rimonabant, and this was accompanied by a marked inhibition of the maximal EGF-induced proliferation of the cells. These authors, however, did not investigate whether increasing endogenous levels of endocannabinoids by blockade of their hydrolysis produced the same results. In Pca tumours, CB_1_ receptor and phosphorylated EGFR immunoreactive scores are positively correlated and provide additive prognostic information with respect to disease-specific survival [8].

The finding that CB_1_ receptor activation mitigates the effects of EGFR in Pca cells [6] is potentially important in therapeutic terms. In the present study, we have investigated the effects of inhibition of 2-AG hydrolysis by JZL184 upon the proliferation and EGFR expression of PC-3 cells.

## Results

### Inter-experimental variation in CB_1_ receptor expression in PC-3 cells

**Figure 1:**
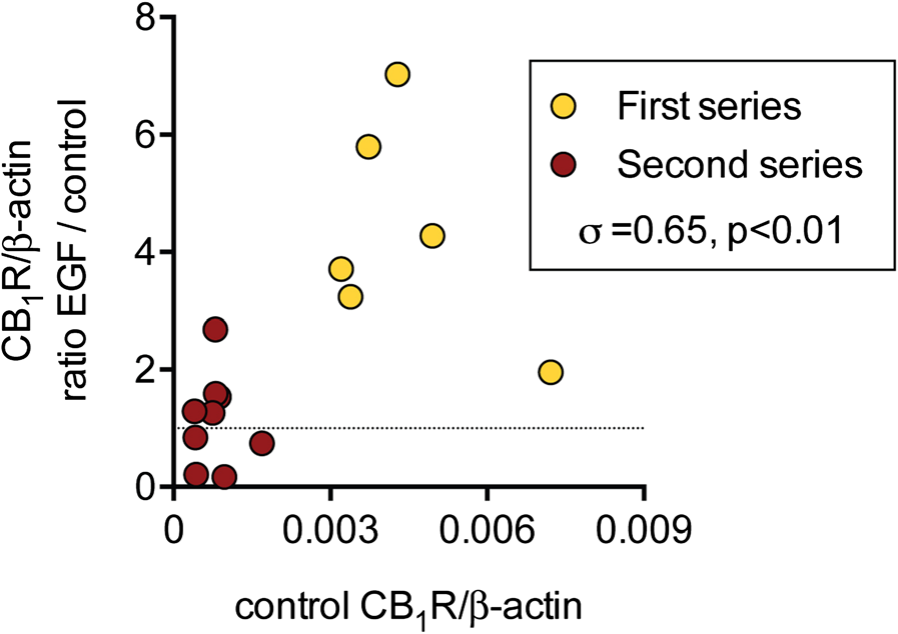
**CB**_**1 **_**receptor expression in PC-3 cells: effect of EGF.** Cells were incubated for 3 weeks in the absence or presence of EGF (10 ng/ml). The x-axis shows the CB_1_ receptor expression, normalised to β-actin for the individual samples, colour coded on the basis of their experimental series. The y-axis shows the CB_1_ receptor expression, normalised to β-actin, in the presence of EGF as a ratio of the expression from another well in the same culture plate cultured in the absence of EGF. The dotted line is at a value of unity, i.e. no stimulation of CB_1_ receptor expression by EGF. The Spearman rho values are shown in the Panel.

Two series of experiments were undertaken using PC-3 cells. The cells were cultured for a total of three weeks without medium change in the absence or presence of EGF (10 ng/ml) (for details, see Methods section). In the first series, a robust expression of CB_1_ receptors was seen. However, in the second series of experiments conducted about half a year later with a new batch of cells but using the same methodology, the levels were very much lower (Figure 1). The cells also behaved differently in their responsiveness to long-term treatment with EGF. For the first experimental series, the EGF treatment increased the observed CB_1_ receptor expression by ~5 fold (median value), whereas no such increase was seen for the second series (Figure 1).

### The influence of EGF upon the expression of sensitivity of PC-3 cells to CP55,940 and JZL184

**Figure 2:**
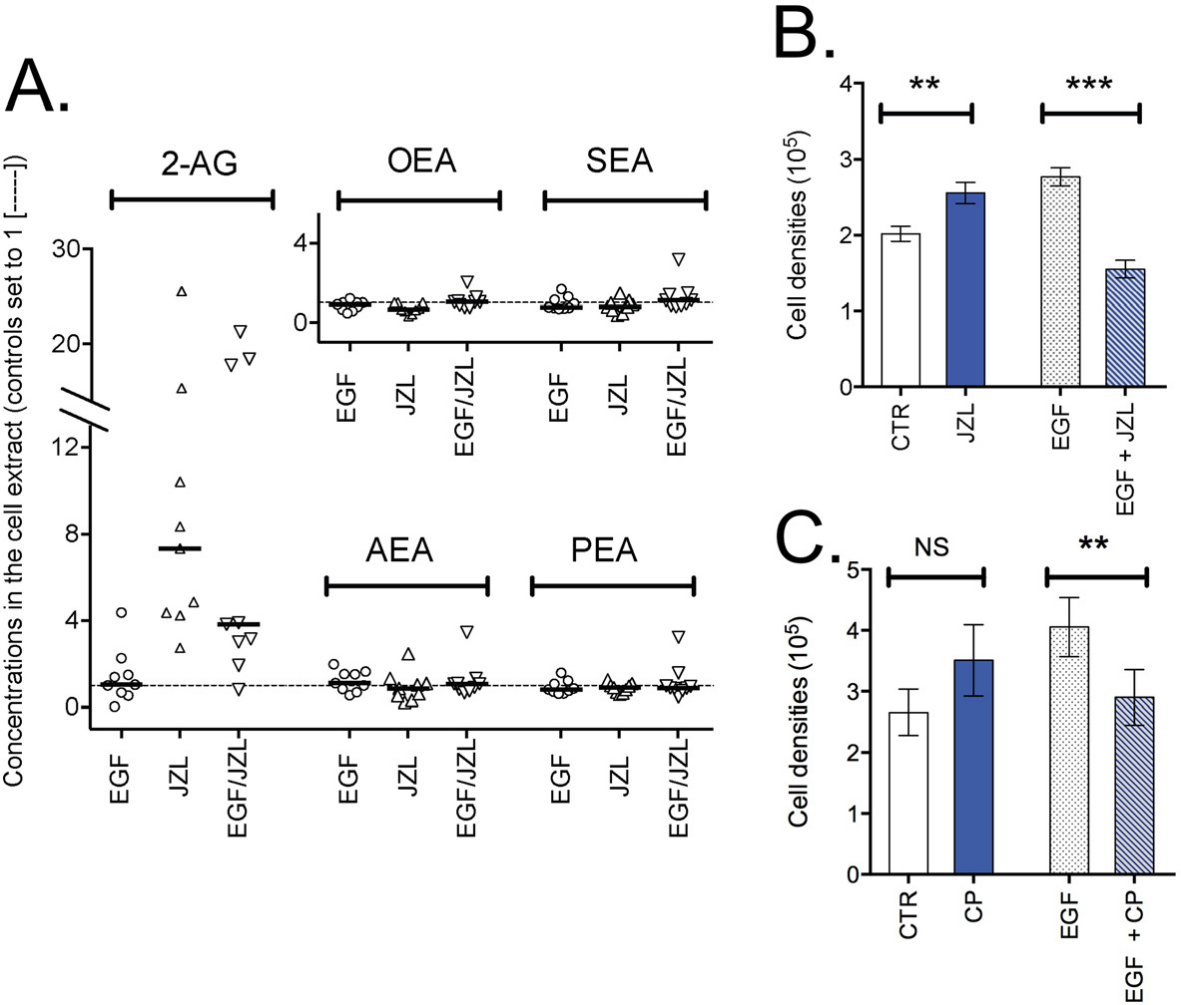
**Effects of JZL184 and CP55,940 upon PC-3 cell proliferation.** Cells were incubated for 3 weeks in the absence or presence of EGF (10 ng/ml). Cells in 6 well plates were treated for three weeks without medium change in the absence or presence of 10 ng/ml of EGF. **A** and **B**: After two of the three weeks, either vehicle or JZL184 (1 μM) was added to the wells and the incubation was continued for a week without change of medium. In **A**, levels of 2-AG and *N*-acylethanolamines are shown. The data, taken for both PC-3 experimental series, show the total levels of the lipids in the cell extract expressed relative to the corresponding value for the extracts taken from wells containing control cells from the same plate. The bars show median values. Abbreviations: 2-AG, 2-arachidonoylglycerol; AEA, anandamide; PEA, palmitoylethanolamide; OEA, oleoylethanolamide; SEA, stearoylethanolamide. In **B** the data are from the first experimental series, and values are means ± s.e.m., n = 6. **P < 0.01, ***P < 0.001, Šídák’s multiple comparison test vs. the corresponding vehicle control following a significant interaction term in the two-way ANOVA matching for JZL184. In **C** (first experimental series), the same experimental protocol was followed, but using 100 nM CP55,940 (“CP”). In **C**, data is given as means ± s.e.m., n = 7 (EGF-treated) or n = 3 (no EGF). **P < 0.01, ^NS^not significant, Šídák’s multiple comparison test vs. the corresponding vehicle control following a significant interaction term in the two-way ANOVA matching for CP55,940.

As part of the first series of experiments with EGF, the cells were treated for the last week with either vehicle or JZL184 (1 μM). The JZL184 treatment produced the expected increase in 2-AG levels without affecting either AEA or related *N*-acylethanolamide levels (Figure 2A). The effect of this compound upon the cell proliferation is shown in Figure 2B. A two-way ANOVA matching for JZL184 indicated a significant interaction JZL184 × EGF (F_1,10_ = 84, P < 0.0001), due to a stimulation of cell proliferation by the compound for control cells and an inhibition for the EGF-treated cells.

**Figure 3:**
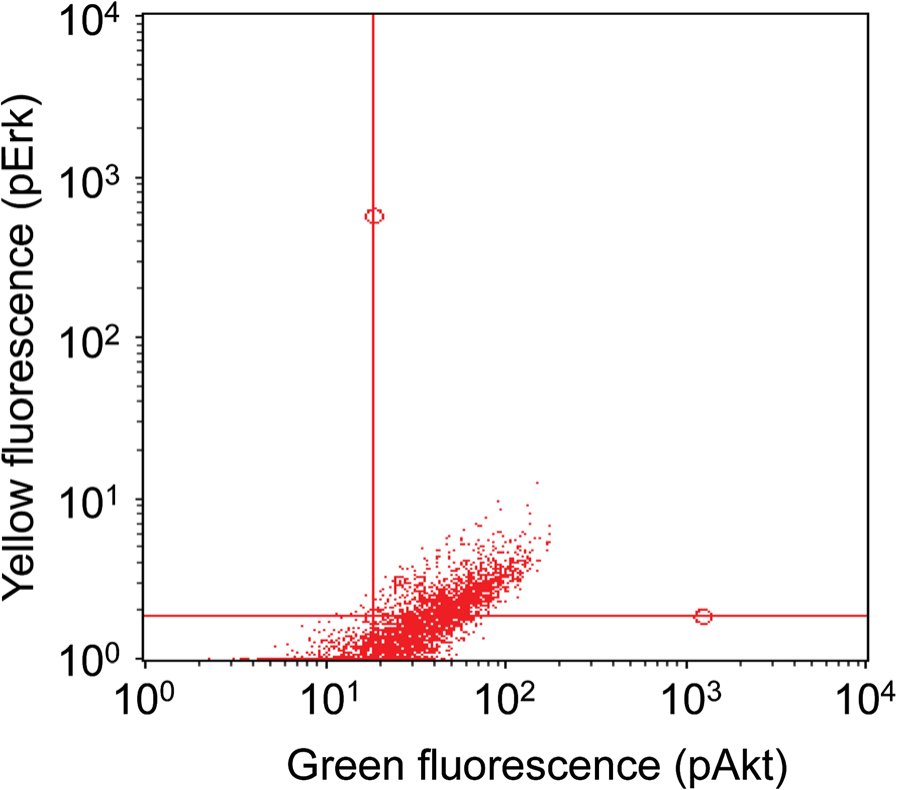
**Cell signalling by PC3 cells.** The figure shows a FACS run for cells treated for a total of three weeks with 10 ng/ml EGF without medium change.

**Figure 4:**
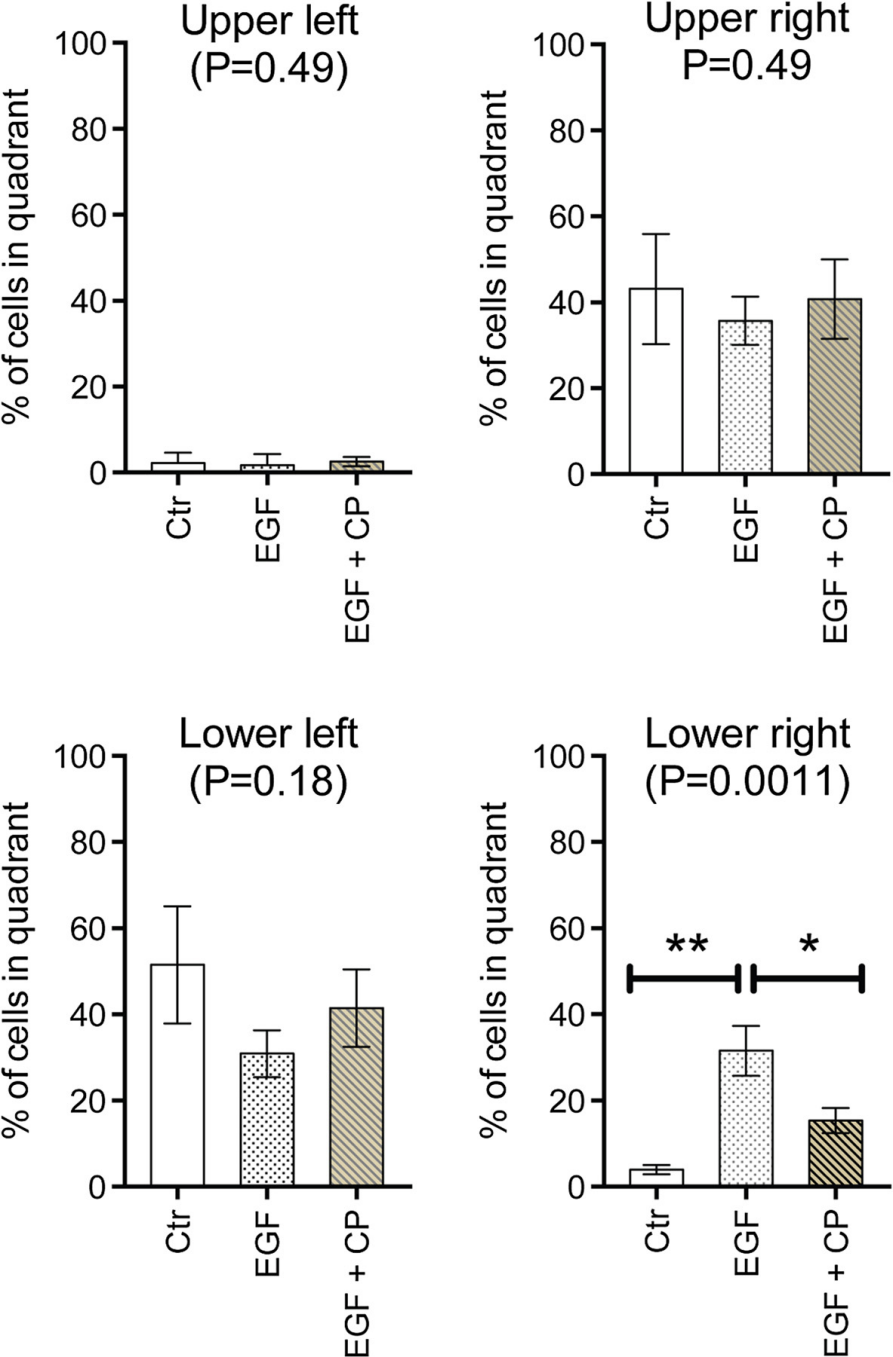
**Effect of EGR and CP55,940 upon the intracellular signalling of human PC-3 prostate cancer cells.** Cells were treated for two weeks without medium change in the absence or presence of 10 ng/ml EGF. Vehicle or 100 nM CP55,940 (“CP”) was then added and the cells were incubated for a further week. Cut-offs (shown by the red lines in Figure 3) were used to define the number and hence % of cells in the four quadrants. (means ± s.e.m., n = 7). P values given in the figures refer to one-way repeated measure ANOVAs not assuming sphericity. **P < 0.01, Tukey’s multiple comparison test following significant ANOVA.

The CB agonist CP55,940 (100 nM) was also investigated in the first series PC-3 cells, although in this case the cells were both counted by FACS and sorted on the basis of their pAkt and pErk immunoreactivities using the FlowCellectTM PI3K/MAPK Dual Pathway Activation and Cancer Marker Detection kit (Merck Millipore, Billerica, MA, USA). An example of the readout for EGF-treated cells is shown in Figure 3. As with JZL184, a significant interaction EGF: CP55,940 was found with the number of cells as endpoint (Figure 2C; two-way ANOVA matching for CP55,940: F_1,8_ (EGF × CP55,940) = 23.65, p < 0.005). The EGF treatment also increased the number of cells in the lower right FACS quadrant following cell sorting (i.e. high pAkt, low pErk expression) and this was also reduced by the CP55,940 treatment (Figure 4). Initial experiments indicated that the inhibition of cell proliferation in the EGF-treated cells by 100 nM CP55,940 was not blocked by the CB_1_ receptor inverse agonist AM251 (100 nM data not shown), suggesting that the effect of CP55,940 at this concentration may be mediated by a non-CB_1_ receptor pathway. Higher concentrations of AM251 were not tested, since they produce rapid anti-proliferative effects in PC-3 cells with low levels of CB_1_ receptor expression [9]. AM251 (admittedly at higher concentrations) also upregulates the mRNA for both EGFR and its ligands in PANC-1 pancreatic cancer cells, which lack CB_1_ receptors [10], so effects (or the lack of them) at higher concentrations of the compound would be very difficult to interpret.

### The influence of JZL184 upon the expression of CB_1_ and EGF receptors

**Figure 5:**
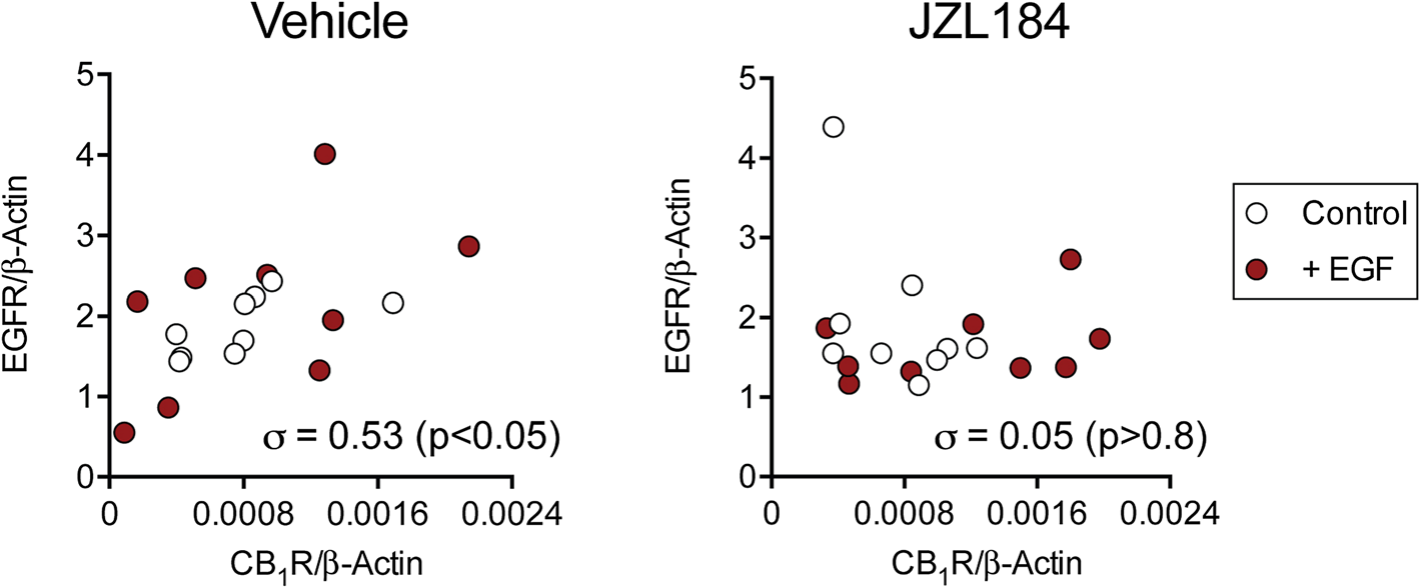
**Effects of EGF and JZL184 upon CB**_**1 **_**receptor and EGFR expression in PC3 cells.** Cells from the second experimental series were incubated with EGF and JZL184 as described in the legend to Figure 2. The EGFR mRNA is shown as a function of the CB_1_ receptor mRNA, both normalised to β-actin, for the second series cells, each data point being for a given well. Shown in each Panel are the Spearman rho values.

For the low CB_1_ receptor-expressing PC-3 cells (i.e. the second experimental series), the effects of EGF and JZL184 upon EGFR expression were investigated (Figure 5) and linear regression analyses were performed. For these analyses, the data for CB_1_ receptor and EGFR expression were logged since residual plots of the regressions using untransformed data were not considered acceptable for parametric analysis (not shown). Using the logged data and with EGFR/β-actin as the dependent variable, the unstandardized coefficients obtained were: EGF, 0.019 ± 0.057, P > 0.7; JZL184, -0.12 ± 0.056, P < 0.05; CB_1_R, 0.31 ± 0.24, P > 0.2; interaction term EGF × CB_1_R, 0.078 ± 0.26, P > 0.7; interaction term JZL184 × CB_1_R, -0.72 ± 0.31, P < 0.05; interaction term EGF × JZL184 × CB_1_R, 0.46 ± 0.36, P > 0.2. The value of the constant was 0.31 ± 0.05 and the ANOVA for the regression was F_6,35_ = 2.89, P < 0.05. Inclusion of the interaction terms in the model increased the adjusted r^2^ value from 0.092 to 0.244.

The finding of a significant interaction CB_1_R × JZL184 allows separate statistical analysis of the untransformed data in the absence or presence of this compound. In the absence of JZL184, a significant correlation between the CB_1_ receptor expression and the EGFR was seen, but this was lost in the presence of JZL184 (Figure 5).

## Discussion and conclusions

The present study was designed to determine whether increasing endogenous 2-AG via MGL inhibition affects the proliferation of EGF-stimulated PC-3 cells secondary to a down-regulation of EGFR expression, consistent with the work of Mimeault and colleagues [6] who used exogenously added AEA as a direct-acting CB_1_ receptor agonist. The protocol we used, whereby cells are incubated for three weeks with EGF without medium change sounds somewhat draconian, but in many ways better represents the situation for the tumour (limited nutrient supply, acidic environment) than standard culturing conditions [11].

The predominant finding of the study is that the expression of CB_1_ receptors in the PC-3 cells varied considerably between the two experimental series. This is not the first time a variation in cell phenotypes has been reported by any means (see [12] and references cited therein), although the only report to our knowledge with respect to the cannabinoid system is from 2003, where we reported that the sensitivity of C6 glioma cells to the anti-proliferative effects of AEA and the 1-regioisomer of 2-AG varied considerably between experiments [13]. This was not due to a solubility issue of these ligands, since the water-soluble analogue phospho-AEA showed the same inter-experimental variation. In contrast, the AEA analogue methanandamide, which inhibited the proliferation in a CB_1_ receptor-independent manner, showed no inter-experimental variability [13]. Variabilities like these and cell responses to other treatments [12] are likely to be under-reported in the literature, but are important with respect to the robustness of the model in question.

The reasons why CB_1_ receptor expression was so different for the two batches of PC-3 cells is unclear, but could be related, for example, to effects on gene transcription induced during the thawing and subsequent culture of the cells. An alternative speculation is that variations in the composition of the foetal serum albumin can affect gene expression. Levels of 2-AG and AEA vary dramatically in different commercial foetal bovine sera batches, and in some cases, this is sufficient to affect cell phenotypes [14]. Whatever the explanation, such a variation can in itself provide a plausible explanation for the differences in CB_1_ receptor-mediated anti-proliferative effects of cannabinoids if it is argued that a threshold level of expression is required for CB_1_ receptor-mediated anti-proliferative effects to occur. Thus, for example, in DU-145 cells, which under the conditions used expressed sufficient CB_1_ receptors to be measurable in radioligand binding experiments, AEA inhibited prolactin-stimulated cell proliferation in a rimonabant-sensitive manner [15]. In contrast, in PC-3 cells with a low expression of CB_1_ receptors under the conditions used, CP55,940 had no effect upon cell proliferation after incubation for up to 6 days [9]. A recent finding from our laboratory that transfection of rat AT1 prostate cancer cells with a murine CB_1_ receptor changes the phenotype from one which responds to nanomolar concentrations of CP55,940 with an increased cell proliferation to one which responds with a reduced cell proliferation [16] would support this conclusion. However, this may be an oversimplification since CB_1_ receptor-independent effects of ∆^9^-tetrahydrocannabinol have been reported in PC-3 cells expressing these receptors [17].

Although the variation seen here is *per se* a complicating factor, some conclusions can be made for the data with the MGL inhibitor JZL184. In the first batch of cells, there is a significant interaction JZL184 × EGF that is also seen for CP55,940 × EGF. At the same time, the EGF treatment increases the expression at the mRNA level of CB_1_ receptors. The simplest explanation of the data are that two processes are occurring: with respect to EGF, the increase in CB_1_ receptor expression changes the phenotype of the cells in the same way as described above for the transfected AT-1 cells. Modest mitogenic effects of low (nanomolar) concentrations of ∆^9^-tetrahydrocannabinol have been reported for PC-3 cells secondary to activation of the phosphoinositide 3-kinase/protein kinase B pathway [18] and a similar process may be occurring here. At the higher CB_1_ receptor expression levels following EGF treatment, JZL184 and CP55,940 are anti-proliferative, suggesting that the cellular pathways involved in these actions (including down-regulation of EGFR, [1,6]) predominate. Evidence in support of this was found with the second batch of cells, despite their low CB_1_ receptor expression. There was a significant interaction between CB_1_R × JZL184 in the linear regression analyses with EGFR expression as the dependent variable, whereas the influence of the EGF treatment was not significant. Analysis of the data suggest that at these low levels of CB_1_ receptor expression, the association of CB_1_ and EGFR is lost in the presence of the MGL inhibitor. This is consistent with the hypothesis that the MGL inhibitor reduces EGFR expression even at low levels of CB_1_ receptor expression.

In conclusion, the data presented here suggest that inhibition of MGL by JZL184 can affect EGFR expression, consistent with the data using AEA to stimulate CB_1_ receptors [6]. However, the use in our hands of PC-3 cells as a model to investigate the therapeutic potential of MGL inhibitors and related compounds is compromised by their variability of CB_1_ receptor expression.

## Methods

### Cell culture and treatments

Human PC-3 prostate cancer cells (passage numbers 3-12 for the experiments reported here) were obtained from DSMZ (Braunschweig, Germany) and cultured in Hams F-10, 2mM L-glutamine, 10% foetal bovine serum and penicillin + streptomycin. Cells (2.5 × 10^5^) were added to 6 well-plates and cultured overnight. The next day, either medium or EGF (final concentration 10 ng/ml) was added, and the cells were cultured for two weeks without change of media. Test compounds (CP55,940 or JZL184 (4-nitrophenyl-4-(dibenzo[d] [1,3] dioxol-5-yl(hydroxy)methyl)piperidine-1-carboxylate, Cayman Chemical Co., Ann Arbor, MI, USA)) were then added, without change of media, and the cells were cultured for a third week. Cells were then harvested and sorted by FACS on the basis of their pAkt and pErk immunoreactivities using the FlowCellectTM PI3K/MAPK Dual Pathway Activation and Cancer Marker Detection kit (Merck Millipore, Billerica, MA, USA).

### RNA extraction, reverse transcription, and qPCR

Cells that were treated with or without EGF and/or JZL184 as described above without medium change were harvested and total RNA was extracted using the miRNeasy Kit (Qiagen, Hilden, Germany) according to the instructions by the manufacturer. The RNA was eluted in 30 μl of RNAse/DNAse-free water and stored at -80°C. The RNA was quantified using a Nanodrop instrument (Thermo Scientific, Wilmington, DE, USA), and cDNA was synthesized from 2 μg of total RNA in 20μl reactions using a cDNA synthesis kit with recombinant moloney murine leukemia virus (rMoMuLV) reverse transcriptase, RNAse inhibitor (1.0 U/μl) and random primers (High capacity cDNA reverse transcription kit; Applied Biosystems, Foster City, CA, USA) according to the instructions by the manufacturer. The mRNA levels for human CB_1_ (*CNR1;* NM_016083), EGFR (NM_005228) and the reference gene β-actin (*ACTB*; NM_001101) were analysed by quantitative real-time PCR (qPCR) using an ABI PRISM® 7900HT Sequence Detection System (Applied Biosystems, Foster City, CA, USA). The qPCR analyses were performed using TaqMan Universal PCR Master Mix and TaqMan gene expression assays including 5′ FAM dye labelled probes (Applied Biosystems, Foster City, CA, USA) for *CNR1* (Assay Hs01038522_s1), *EGFR* (Assay Hs01076078_m1), and *ACΤΒ* (Assay Hs00357333_g1). The sample volume was 10 μl per well (384 well format) and the qPCR running protocol as follows: Initial steps at 50°C for 2 min and 95°C for 10 min, followed by 40 cycles of denaturation at 95°C for 15 sec and annealing/extension at 60°C for 1 min. No amplification was detected in control samples where cDNA template was omitted (data not shown). The relative expression of CB_1_ and EGFR was normalized with β-actin used as reference gene according to the ΔΔC_q_ method.

### Measurement of 2-AG, AEA and related *N*-acylethanolamines in cell extracts

The lipids were extracted as described by Bradshaw *et al.*[19]. Briefly, after removal of medium, the cells were washed twice with phosphate-buffered saline after which methanol (2 ml) was added. The mixture was scraped using a rubber policeman and the extract pipetted into Falcon tubes. An additional 1 ml of methanol was added to the wells, the wells scraped and the mixture was pipetted into the same tubes. These were then centrifuged at 2000 × g to sediment cell debris, and the methanol phase collected. Water was added to give a final methanol concentration of 30% (v/v). 2-AG, AEA and related lipids in the extracts were analysed as described elsewhere [20]. Briefly, the cell extracts spiked with internal standard (AEA-d8, 2-AG-d8 and OEA-d4) were subjected to solid phase extraction (SPE) on 200 mg Waters Oasis HLB cartridges (Milford, MA, USA). After evaporation of the SPE elutions using a MiniVac system (Farmingdale, NY, USA), the analytes were reconstituted in 100 μL of methanol. Ten μL of recovery standard (DHEA-d4) was added and ultra performance liquid chromatography coupled with electrospray ionization tandem mass spectrometry (UPLC-ESI-MS/MS) analysis was performed immediately. A Waters Acquity Ultra Performance (Milford, MA, USA) equipped with a 2.1 mm × 150 mm Waters BEH C18 column with a 2.5 μm particle size was used for UPLC with the following conditions: the autosampler was kept at 10°C, mobile phase A was water, mobile phase B was methanol with 10 mM ammonium acetate. Gradient elution was performed at a flow rate of 0.4 mL/min. The column was connected to a Waters triple quadrupole MS (Micromass Quattro Ultima) equipped with an ESI source operating in positive multiple reaction monitoring (MRM) mode. The optimized conditions and the MRM transitions, as well as extraction efficiencies have been reported in [20]. MassLynx software was used to quantify the peaks according to the stable isotope dilution method.

### Statistics

Two statistical software programmes were used. ANOVA and Spearman’s correlation coefficients were determined using the statistical package built into the GraphPad Prism computer programme for the Macintosh (GraphPad Software Inc., San Diego, CA, USA). The linear regression analyses were undertaken using the IBS SPSS Statistics package, version 22.

## Competing interests

The authors declare that they have no competing interests.

## Authors’ contributions

MC was involved in the conception of the study and undertook the cell culturing experiments. SG-F and MN were responsible for the analyses of the endocannabinoids and related lipids. EP was responsible for the mRNA measurements. CJF was involved in the conception of the study, the analyses and wrote the paper. All authors read and approved the final manuscript.
